# Nonalcoholic Fatty Liver Disease, Liver Fibrosis, and Utility of Noninvasive Scores in Patients With Acromegaly

**DOI:** 10.1210/clinem/dgad490

**Published:** 2023-08-17

**Authors:** İmdat Eroğlu, Burcin Gonul Iremli, Ilkay S Idilman, Deniz Yuce, Incilay Lay, Deniz Akata, Tomris Erbas

**Affiliations:** Department of Internal Medicine, Hacettepe University, School of Medicine, 06230, Ankara, Turkey; Department of Internal Medicine, Hacettepe University, School of Medicine, 06230, Ankara, Turkey; Department of Endocrinology and Metabolism, Hacettepe University, School of Medicine, 06230, Ankara, Turkey; Department of Radiology, Hacettepe University, School of Medicine, 06230, Ankara, Turkey; Department of Preventive Oncology, Hacettepe University, School of Medicine, 06230, Ankara, Turkey; Department of Biochemistry, Hacettepe University, School of Medicine, 06230, Ankara, Turkey; Department of Radiology, Hacettepe University, School of Medicine, 06230, Ankara, Turkey; Department of Internal Medicine, Hacettepe University, School of Medicine, 06230, Ankara, Turkey; Department of Endocrinology and Metabolism, Hacettepe University, School of Medicine, 06230, Ankara, Turkey

**Keywords:** acromegaly, growth hormone, NAFLD, MRI-PDFF, MRE-LSM, ANGPTL-8, noninasive scores

## Abstract

**Context:**

Nonalcoholic fatty liver disease (NAFLD) is a metabolical disorder and can lead to liver fibrosis. Because it is commonly seen, several noninvasive scores (NS) have been validated to identify high-risk patients. Patients with NAFLD have been shown to have higher serum angiopoietin-like protein-8 (ANGPTL-8) levels.

**Objective:**

The risk of NAFLD is known insufficiently in acromegaly. Moreover, the utility of the NS and the link between NAFLD and ANGPTL-8 in acromegaly is unknown.

**Methods:**

Thirty-two patients with acromegaly (n = 15, active [AA] and n = 17, controlled acromegaly [CA]) and 19 healthy controls were included. Magnetic resonance imaging (MRI)-proton density fat fraction (PDFF) was used to evaluate hepatic steatosis, and magnetic resonance elastography to evaluate liver stiffness measurement. ANGPTL-8 levels were measured with ELISA.

**Results:**

Median liver MRI-PDFF and NAFLD prevalence in AA were lower than in CA (*P* = .026 and *P* < .001, respectively). Median magnetic resonance elastography-liver stiffness measurement were similar across groups. Of the NS, visceral adiposity index, fatty liver index, hepatic steatosis index, and triglyceride-glucose index (TyG) all showed positive correlation with the liver MRI-PDFF in the control group. However, only TyG significantly correlated with liver fat in the AA and CA groups. There was no correlation between traditional NAFLD risk factors (body mass index, waist circumference, C-reactive protein, homeostasis model assessment for insulin resistance, visceral adipose tissue) and liver MRI-PDFF in the AA and CA. Patients with acromegaly with NAFLD had lower GH, IGF-1, and ANGPTL-8 levels than in those without NAFLD (*P* = .025, *P* = .011, and *P* = .036, respectively).

**Conclusion:**

Active acromegaly may protect from NAFLD because of high GH. In patients with acromegaly, NAFLD risk cannot be explained with classical risk factors; hence, additional risk factors must be identified. TyG is the best score to evaluate NAFLD risk. Lower ANGPTL-8 in patients with acromegaly and NAFLD implies this hormone may be raised because of insulin resistance rather than being a cause for NAFLD.

Nonalcoholic fatty liver disease (NAFLD) is a major health problem worldwide and is recognized as the hepatic component of metabolic syndrome. NAFLD can lead to fibrosis, cirrhosis, and hepatocellular carcinoma (1). Although NAFLD is most typically connected with diabetes mellitus and obesity, it is also associated with several endocrinopathies (2). Acromegaly is characterized by increased secretion of GH. In acromegaly, glucose and lipid metabolisms are disrupted, the amount of free fatty acids in circulation rises, and, as a result, hepatic and extrahepatic insulin resistance and alterations in fat distribution develop. However, increasing GH levels can also cause enhanced lipolysis and reduced total body fat.

The relation between acromegaly and NAFLD remains unclear. Several studies have indicated that people with active acromegaly had less liver fat, and biochemical control of the acromegaly after surgery increases intrahepatic lipid content (3-7). Moreover, visceral adipose tissue and intrahepatic lipid content were not independent predictors of the active acromegaly and both were increased after surgery (8). Conversely, patients with acromegaly were shown to have a higher hepatic steatosis index, which improved along with disease control (9). Patients with acromegaly have a unique pattern of body composition called acromegaly-specific lipodystrophy (10). So, understanding how lipodystrophy develops and how different treatments for acromegaly change its symptoms is essential for improving the long-term health of people with this disease.

Although biopsy is the gold standard for assessing the degree of hepatosteatosis and fibrosis, because it is not routinely applicable, magnetic resonance imaging (MRI) and elastography (MRE) can provide information regarding liver fat ratio and parenchymal stiffness (11). Because of the high cost and interobserver differences of imaging techniques, several noninvasive scores have been developed to identify high-risk individuals in non-acromegalic populations. Fatty liver index (FLI), hepatic steatosis index (HSI), triglyceride-glucose index (TyG), and visceral adiposity index (VAI) are scores used to predict the existence of NAFLD (12-16). Furthermore, BARD, aspartate aminotransferase/platelet ratio index (APRI), fibrosis-4 score (FIB-4), and NAFLD fibrosis score (NFS) are validated scores for evaluating the risk of fibrosis (17, 18). Many serum biomarkers have been explored for use in the diagnosis and follow-up of NAFLD, with serum angiopoietin-like protein-8 (ANGPTL-8) levels being one of the most promising. Many studies including nonacromegalic populations have indicated that serum ANGPTL-8 levels are higher in patients with NAFLD (19-21).

Although noninvasive scores and serum ANGPTL-8 levels have been investigated in patients without acromegaly but with NAFLD, their utility in acromegaly remains uncertain. In this study, we evaluated the risk of NAFLD and hepatic fibrosis in patients with acromegaly and their association with the noninvasive scores and ANGPTL-8 levels for the first time in the literature.

## Patients and Methods

### Study Design and Participants

This study was conducted between September 2021 and June 2022 at the Hacettepe University, School of Medicine, Department of Endocrinology and Metabolism. This study was approved by the Ethics Committee of Hacettepe University (Project Number: GO 21/166, Decision Number:2021/05-38). The study was performed in accordance with the Declaration of Helsinki. Written informed consent was obtained from all of the subjects. Thirty-two patients with acromegaly (15 active [AA] and 17 controlled acromegaly [CA]) and 19 age, gender, and body mass index (BMI)-matched healthy controls were included. To minimize selection bias, individuals who had a clinical or radiological suspicion of NAFLD at any time in their lives were not included in the control group, but individuals who were determined to have NAFLD during the research were not excluded either.

The AA group (n = 15) consisted of newly diagnosed patients or patients with high IGF-1 levels according to the reference points determined by age and gender. The CA group (n = 17) included patients whose IGF-1 levels were within the normal range according to age- and sex-determined references at 3 visits minimum.

### Excluding Criteria

Individuals <18 and >65 years of age, pregnant and lactating women, those with chronic alcohol use (>20 g/day for women, >30 g/day for men), individuals with active viral hepatitis and another liver disease with established etiology, active patients with cancer, individuals with chronic kidney disease, cardiopulmonary failure, those with a previous diagnosis of rheumatological disease or who were treated with biologic agents or chemotherapy for any reason before, and individuals receiving glucocorticoid therapy for a reason other than hypocortisolism were excluded from the study. All individuals diagnosed with diabetes mellitus in the control group were excluded from the study. Because acromegaly is a rare disease, and mild to moderate dysglycemia secondary to high GH and IGF-1 exposure is very common in patients with acromegaly (22, 23), we did not exclude patients with prediabetes and diabetes in the acromegaly group. However, to eliminate the devastating effects of severe diabetes mellitus, we did not include patients with acromegaly using insulin.

### Laboratory Analysis

Hormonal and biochemical parameters were measured in the early morning following at least 8 hours of overnight fasting. Oral glucose tolerance tests (75 g) were done to measure all of the participants’ glucose and insulin levels at 0 and 120 minutes. Insulin resistance was determined using the homeostasis model assessment for insulin resistance (HOMA-IR) index (24). To rule out the possibility of underlying secondary liver disease, viral serology (including for hepatitis A, B, and C), autoimmune hepatitis markers (including antinuclear antibodies, antimitochondrial antibodies, antismooth muscle antibodies, liver-kidney microsomal antibodies), and ceruloplasmin and transferrin saturation levels were also measured. A chemiluminescence microparticle enzyme immunoassay (Siemens Healthineers, Immulite 2000 XPi Immunoassay System, Germany) method was used for GH measurement and immunoradiometric assay (DSL-2800 ACTIVE, Diagnostic System Laboratories Inc., Texas, USA) was used for the measurement of IGF-1. For other biochemical methods, see the supplemental information (25).

### Measurement of Angiopoietin-like Protein-8

After at least 8 hours of fasting, the participants’ serum part of the blood samples taken early in the morning was separated by centrifugation and stored at −80 °C. Human ANGPTL-8 ELISA Kit (Cloud-Clone Corp Cat# SEW803Hu, RRID:AB_2938597) was used for measurement. Intra-assay and inter-assay coefficients of variability were <10% and <12%, respectively. The kits were studied in accordance with the manufacturer's instructions by applying a 1:20 dilution to the serum samples.

### Noninvasive Hepatosteatosis and Fibrosis Scores

Noninvasive hepatosteatosis and fibrosis scores were calculated to identify hepatosteatosis and fibrosis risk (supplemental information) (25). VAI, FLI, HSI, and TyG were calculated as noninvasive hepatosteatosis scores. In terms of adipose tissue dysfunction, participants were put into one of 4 groups based on their VAI values and the cutoffs that had already been set in the literature (26). FLI values <30 were considered low risk for NAFLD, >60 high risk, and values in between were considered as indeterminate NAFLD risk (13). Those with an HSI score of <30 were considered at low risk of NAFLD, those with >36 were considered at high risk of NAFLD, and values between 30 and 36 were considered uncertain in terms of NAFLD risk (14). Participants having a TyG score of 8.5 or above were regarded to have a high risk for NAFLD (16).

Noninvasive fibrosis scores including NFS, BARD, APRI, and FIB-4 were calculated. Individuals with an NFS score of −1.455 were considered to have a low suspicion of fibrosis, participants with a score of >0.675 were thought to have a high suspicion of fibrosis, and participants with a score between these values were thought to be uncertain for fibrosis risk (27). Patients with an APRI score of <0.5 were considered to have ruled out fibrosis, those between 0.5 and 0.7 had some kind of liver damage, those between 0.7 and 1 had significant fibrosis, and those with >1 were associated with cirrhosis (28). According to the BARD score, 0 to 1 points were considered to have low risk and 2 to 4 points high risk in terms of severe fibrosis risk (29). Those with a FIB-4 score <1.45 were considered to have low risk for cirrhosis, those with a FIB-4 score >3.25 were considered to be at high risk for cirrhosis, and those with intermediate values were considered to be uncertain in terms of cirrhosis risk (30).

### Magnetic Resonance Imaging

#### Imaging protocol

All participants (except for 1 active patient with acromegaly) underwent liver MRI with a 1.5-T system (Siemens AERA, Germany) with standard body and spine matrix coils. The multi-echo Dixon method was used with a VIBE sequence (Siemens Healthcare) to evaluate MRI proton density fat fraction (PDFF) with the following parameters: repetition time 15.6 ms, 6 echo times (1.23, 2.48, 3.73, 4.98, 6.23, and 7.48 ms), flip angle 4 degrees, readout echo bandwidth 1080 Hz/pixel, field of view 450 mm, and slice thickness 3.5 mm. MRE was performed with a 2-dimensional gradient-recalled echo sequence with the active driver generating waves at 60 Hz with the following parameters: repetition time/echo time, 50/21 ms; flip angle: 25 degrees; bandwidth: 31.25 kHz; matrix: 256 × 128; and acquisition time: 2.5 minutes. Depending on the liver size, 2 or 3 slices of 10-mm thickness were obtained from the largest part of the liver by the patient holding his or her breath.

#### Imaging analysis

All MRI and MRE measurements were performed by using a workstation (Syngo.via VB10; Siemens Medical Solutions). All measurements were performed by a single radiologist (I.S.I.), who has 16 years of experience. MRI-PDFF were measured from 4 different slices by drawing as large as possible region of interest (ROI) placed over the liver by excluding lesions, large vessels, liver margins, and artifacts from the right lobe of the liver and average was calculated. Liver stiffness measurements (LSM) were performed by drawing freehand ROIs as geographic areas guided by the magnitude image to include liver parenchyma by excluding major vessels. Measurements were repeated on the confidence map images.

The opposed-phase image was chosen as the India-ink artifact between the fat and water interface and helped in the demarcation of the extent of the visceral adipose tissue (VAT) and subcutaneous adipose tissue. VAT and subcutaneous adipose tissue were measured by a freehand ROI and expressed as cm^2^ from the level of L3 vertebra.

#### Definition of the severity of NAFLD and hepatic fibrosis

NAFLD was diagnosed in individuals with a liver MRI-PDFF ≥5%. The subjects were then categorized as follows: <5%, normal liver; 5% to 14%, mild fatty liver; 14% to 28%, moderate fatty liver; and >28%, severe fatty liver (31). Those with MRE-LSM <2.5 kPa were considered normal and those with ≥2.5 kPa were accepted as increased MRE-LSM. For the definition of diabetes mellitus, hypertension, and dyslipidemia see supplemental material (25).

#### Statistical analysis

Statistical analyzes were performed with IBM SPSS software, version 25.0. Categorical variables are shown as numbers and percentages. Because the numerical variables do not follow the normal distribution, the median and interquartile range (25th percentile-75th percentile) are given. Normal distribution was evaluated with the Kolmogorov-Smirnov test. Correlation coefficients (*r*) and statistical significance (*P*) were calculated with Spearman's test for the relationships between numerical variables, at least 1 of which was not normally distributed. Statistical significance value was determined as *P* < .05. Active and controlled acromegaly groups were considered as a single “acromegaly” group for receiver operating characteristic analyses. In correlation analysis for absolute values of *r*, 0 to 0.19 is considered extremely weak, 0.2 to 0.39 is considered weak, 0.40 to 0.59 is considered moderate, 0.6 to 0.79 is considered strong, and 0.8 to 1 is considered very strong (32).

## Results

### Characteristics of the Active and Controlled Patients With Acromegaly

Of the 32 patients with acromegaly, 15 had AA and 17 had CA. Duration of the disease was longer in those with CA than AA (*P* = .047). Four (26.6%) patients in the AA group had diabetes mellitus; 2 were followed only with diet, 1 was on metformin + vildagliptin, and 1 was on pioglitazone therapy. On the other hand, 5 (29.4%) patients in the CA group had diabetes; 3 were followed only with diet and 2 were on metformin monotherapy. Other features and current treatments of the AA and CA group were shown in Table 1.

**Table 1.** Clinical characteristics of active and controlled patients with acromegaly

Variable	Active acromegaly	Controlled acromegaly	*P* value
	(n = 15)	(n = 17)	
Disease duration, y	5.9	12.3	**.047**
	[3.3-11.7]	[8.2-14]	
Surgical history (n, %)			
Unoperated	5 (33.3%)	—	**.038**
Operated	10 (66.6%)	17 (100%)	
Radiotherapy (n, %)			
Received	—	5 (29.4%)	**.046**
Not received	15 (100%)	12 (70.5%)	
Medical treatment (n, %)			
Following without treatment	3 (20%)	4 (23.5%)	.083
Newly diagnosed	4 (26.6%)	—	
SRL	5 (33.3%)	11 (64.7%)	
SRL + cabergoline	2 (13.3%)	1 (5.8%)	
SRL + pegvisomant	1 (6.6%)	—	
SRL + pegvisomant + cabergoline	—	1 (5.8%)	
SRL treatment (n, %)	8 (53.3%)	12 (70.6%)	.314
Pegvisomant treatment (n, %)	1 (6.7%)	1 (5.9%)	1
Cabergoline treatment (n, %)	1 (6.7%)	2 (11.8%)	1
Antidiabetic treatment (n, %)	2 (13.3%)	3 (17.6%)	1
Antilipemic treatment (n, %)	1 (6.7%)	2 (11.8%)	1
Antihypertensive treatment (n, %)	4 (26.7%)	7 (41.2%)	.388
Hormone replacement (n, %)			
L-thyroxine	5 (33.3%)	5 (29.4%)	1
Glucocorticoid	—	2 (11.7%)	.486
Testosterone/ Estrogen	—	4 (23.5%)	.104

Median [25 percentile-75 percentile]. The boldface *P* values indicate statistical significance at the *P* ≤ .05 level.

Abbreviation: SRL, somatostatin receptor ligand.

### Active Acromegaly, Controlled Acromegaly, and the Control Groups

#### Demographic, anthropometric, clinical, and biochemical characteristics

Age, gender, and BMI distributions were similar across the groups. Waist circumference was significantly higher in the CA group compared with the control group (*P* = .047). AA had significantly higher IGF-1 levels than CA and the control group (*P* < .001 and *P* < .001, respectively), whereas CA had significantly higher IGF-1 levels than the control group (*P* = .030). The median ANGPTL-8 level in CA was significantly lower than in AA (*P* = .006), whereas it tended to be lower than the control group (*P* = .068). Other hormonal and metabolic parameters of the groups are shown in Table 2.

**Table 2.** Demographic, anthropometric, clinical, and biochemical characteristics of the participants

Variable	Active acromegaly (n = 15)	Controlled acromegaly (n = 17)	Control group(n = 19)	*P*-1*^a^*	*P*-2*^a^*	*P*-3*^a^*
Gender (F/M)	6/9	9/8	9/10	.67	.74	.46
Body mass index (kg/m^2^)	29.6 [27–30.9]	31.6 [27.6–32.1]	29.4 [27.7–31.6]	.639	.375	.345
Waist circumference (cm)	94 [86–101]	103 [97–106]	94 [89–103]	.795	.**047**	.059
Hip circumference (cm)	108 [106–114]	114 [107–117]	112 [105–116]	.487	.308	.072
Waist/hip ratio	0.85 [0.81–0.9]	0.91 [0.85–0.94]	0.84 [0.79–0.91]	.665	.099	.234
Hypertension (n, %)	5 (33.3%)	7 (41.1%)	5 (26.3%)	.71	.35	.65
Hyperlipidemia (n,%)	8 (53.3%)	13 (76.4%)	8 (42.1%)	.52	.**037**	.17
Hemoglobin (gr/dL)	14.4 [13.2–15.5]	13.7 [12.6–14.5]	14.6 [13.7–15.5]	.755	.**032**	.100
C-reactive protein (mg/dL)	0.25 [0.13–0.39]	0.36 [0.22–0.52]	0.4 [0.24–0.5]	.099	.949	.108
ALT (U/L)	16 [12–22]	20 [14–27]	21 [17–29]	.**040**	.302	.167
AST (U/L)	19 [15–21]	19 [17–29]	20 [18–25]	.223	.679	.569
Fasting plasma glucose (mg/dL)	102 [89–124]	107 [98–110]	100 [89–106]	.298	.059	.692
Fasting insulin (µIU/mL)	8 [5.54–10.16]	5.9 [4.09–12.94]	6.17 [4.44–7.27]	.077	.680	.606
HOMA-IR	1.8 [1.4–3]	1.6 [1.1–3.6]	1.3 [0.8–1.8]	.**05**	.204	.558
Total cholesterol (mg/dL)	182 [163–220]	238 [218–253]	189 [180–218]	.110	.**014**	.**005**
LDL (mg/dL)	114 [105–142]	159 [133–168]	122 [118–144]	.077	.**014**	.**009**
HDL (mg/dL)	51 [39–58]	49 [40–59]	49 [41–63]	.603	.849	.734
Triglyceride (mg/dL)	93 [81–117]	141 [115–213]	84 [65–116]	.314	.**003**	.**006**
25-OH-vitamin D (µg/L)	19 [10.89–26.85]	17.11 [7.91–22.29]	17.41 [6.4–26.18]	.563	.843	.558
TSH (uIU/mL)	1.82 [1.14–2.75]	1.75 [1.15–2.39]	1.94 [1.41–3.17]	.435	.350	.777
ACTH (pg/mL)	24.7 [17.1–41.7]	24.6 [20.5–50]	15.7 [13.5–25.4]	.**030**	.**01**	.756
Cortisol (µg/dL)	10.21 [9.52–12.26]	10.37 [8.56–13.1]	12.12 [8.91–13.28]	.755	.849	.855
FSH (mIU/mL)	5.15 [3.47–13.11]	5.69 [3.09–12.27]	5.69 [3.24–7.63]	.822	.727	.880
LH (mIU/mL)	3.11 [1.67–6.69]	2.34 [0.78–4.72]	3.8 [1.89–5.55]	.742	.145	.282
Estradiol (pg/mL) (for females)	19 [12–86]	21 [20.86–23.76]	33 [18–50]	.637	.508	.679
Testosterone (ng/dL) (for males)	368.58 [357.99–413.13]	280.65 [230.16–498.82]	373.17 [325.35–723.87]	.722	.214	.529
IGF-1 (ng/mL)	493.8 [371.5–771.1]	232.1 [153.4–277.8]	159.5 [105–207.4]	**<.001**	.**030**	**<.001**
ANGPTL-8 (ng/mL)	0.89 [0.69–1.2]	0.61 [0.53–0.7]	0.67 [0.62–0.92]	.140	.068	.**006**

Median [25 percentile-75 percentile]. The boldface *P* values indicate statistical significance at the *P* ≤ .05 level.

Abbreviations: ALT, alanine aminotransferase; ANGPTL-8, angiopoietin like protein-8; AST, aspartate aminotransferase; F, Female; HDL, high-density lipoprotein; HOMA-IR, homeostasis model assessment index-insulin resistance; LDL, low-density lipoprotein; M, male.

^*a*^ *P*-1, active acromegaly vs control group; *P*-2, controlled acromegaly vs control group; *P*-3, active acromegaly vs controlled acromegaly.

#### NAFLD prevalence, liver MRI-PDFF, and noninvasive hepatosteatosis scores of the Participants

The median liver MRI-PDFF was 1.75% (1.4%-2.6%) in AA, 6.5% (2%-9.2%) in CA, and 3.5% (2%-6.6%) in the control group. Although liver MRI-PDFF was significantly lower in AA than in CA (*P* = .026), it tended to be lower in AA than in the control group. No significant difference was observed between CA and the control group for liver MRI-PDFF. None of the patients in AA was diagnosed with NAFLD, whereas 58.8% (n = 10) of the patients in CA and 26.3% (n = 5) of the control group were diagnosed as NAFLD. The prevalence of NAFLD was found to be significantly higher in CA than in AA (*P* < .001). The prevalence of NAFLD tended to be lower in AA compared with the control group (*P* = .057). Moreover, VAT measured by MRI-PDFF was also found to be lower in AA than in CA and control group (Table 3).

**Table 3.** Noninvasive scores, liver fat fraction, and parenchymal stiffness of the participants

Variable	Active acromegaly (n = 15)	Controlled acromegaly (n = 17)	Control group	*P*-1*^a^*	*P*-2*^a^*	*P*-3*^a^*
			(n= 19)			
HSI	37.6	39	40	.089	.862	.174
	[35.4–39.9]	[36.5–42.1]	[37–41.3]			
HSI risk (n, %)						
Low	—	—	1 (5.2%)	1	1	1
Indeterminate	4 (26.6%)	4 (23.5%)	4 (21%)			
High	11 (73.3%)	13 (76.4%)	14 (73.6%)			
FLI	38	69	49	.386	**.029**	**.03**
	[25–70]	[52–84]	[33–66]			
FLI risk (n, %)						
Low	4 (26.6%)	2 (11.7%)	2 (10.5%)	.53	.086	.06
Indeterminate	7 (46.6%)	3 (17.6%)	10 (52.6%)			
High	4 (26.6%)	12 (70.5%)	7 (36.8%)			
TyG index	8.5	9.04	8.36	.314	**.001**	**.022**
	[8.14–8.92]	[8.83–9.32]	[8.15–8.61]			
Visceral adiposity index	1.21	2.28	1.08	.267	**.005**	**.03**
	[0.94–2.06]	[1.59–3.56]	[0.8–1.88]			
Adipose tissue dysfunction (n, %)						
Absent	12 (80%)	7 (41.1%)	17 (89.4%)	.85	**.004**	.17
Mild	1 (6.6%)	2 (11.7%)	1 (5.2%)			
Moderate	1 (6.6%)	3 (17.6%)	1 (5.2%)			
Severe	1 (6.6%)	4 (23.5%)	—			
TyG index risk						
Low NAFLD risk	7 (46.6%)	2 (11.7%)	14 (73.6%)	.11	**<.001**	**.049**
High NAFLD risk	8 (53.3%)	15 (88.2%)	5 (26.3%)			
Visceral adipose tissue (cm^2^)	43.92	101.3	87.97	**.029**	.303	**.005**
	[28.97–90.73]	[90.15–151.29]	[63.7–163.62]			
Liver MRI-PDFF (%)	1.75	6.5	3.5	.071	.21	**.026**
	[1.4–2.6]	[2–9.2]	[2–6.6]			
NAFLD (n, %)	—	10 (58.8%)	5 (26.3%)	.057	.09	**<**.**001**
Hepatosteatosis severity (n, %)						
Absent	14 (100%)	7 (41.1%)	14 (73.6%)	.057	.09	**.001**
Mild	—	9 (52.9%)	5 (26.3%)			
Moderate	—	1 (5.8%)	—			
Severe	—	—	—			
NFS	−1.53	−1.81	−1.9	.176	.288	.417
	[−1.89–−0.04]	[−2.4–−0.57]	[−2.7–−0.92]			
NFS risk (n, %)						
Low	8 (53.3%)	10 (58.8%)	13 (68.4%)	.37	.55	1
Indeterminate	7 (46.6%)	7 (41.1%)	6 (31.5%)			
High	—	—	—			
APRI	0.19	0.21	0.24	.543	.546	.865
	[0.14–0.33]	[0.14–0.28]	[0.16–0.28]			
APRI risk (n, %)						
No fibrosis	15 (100%)	17 (100%)	19 (100%)	—	—	—
Mild liver injury	—	—	—			
Severe fibrosis	—	—	—			
Cirrhosis risk	—	—	—			
BARD	3	3	2	**.018**	.17	.23
	[2–4]	[2–3]	[1–3]			
BARD risk (n, %)						
Low	—	2 (11.7%)	6 (31.5%)	**.02**	.24	.47
High	15 (100%)	15 (88.2%)	13 (68.4%)			
FIB-4	0.9	0.97	0.86	.521	.536	.985
	[0.71–1.19]	[0.56–1.35]	[0.57–1.09]			
FIB-4 risk (n. %)						
Low	12 (80%)	11 (64.7%)	16 (84.2%)	1	.26	.441
Indeterminate	3 (20%)	6 (35.2%)	3 (15.7%)			
High	—	—	—			
Liver stiffness measurement (kPa)	2.31	2.29	2.17	.316	.216	.984
	[2.07–2.5]	[2.11–2.41]	[1.97–2.43]			
Increased LSM (n, %)						
Absent	11 (78.5%)	13 (76.4%)	17 (89.4%)	.63	.39	1
Present	3 (21.4%)	4 (23.5%)	2 (10.5%)			

Median [25 percentile-75 percentile]. The boldface *P* values indicate statistical significance at the *P* ≤ .05 level.

Abbreviations: APRI, aspartate aminotransferase/platelet ratio index; FIB-4, fibrosis-4 score; FLI, fatty liver index; HSI, hepatic steatosis index; LSM, liver stiffness measurement; MRE-LSM, magnetic resonances elastography liver stiffness measurement; MRI-PDFF, magnetic resonances imaging proton density fat fraction; NAFLD, nonalcoholic fatty liver disease; NFS, NAFLD fibrosis score; TyG, triglyceride-glucose index.

^*a*^ *P*-1, active acromegaly vs control group; *P*-2, controlled acromegaly vs control group; *P*-3, active acromegaly vs controlled acromegaly.

Of the noninvasive hepatosteatosis score, HSI was similar between groups; however, FLI and TyG were considerably greater in CA compared with the other groups. The number of individuals with high risk of NAFLD according to both scores was higher in the CA than in the AA and controls. The CA group had a higher VAI. Although there was no one with severe adipose tissue dysfunction in the control group, severe adipose tissue dysfunction was detected in 1 (6.6%) patient in the AA group and 4 (23.5%) patients in the CA. This difference was significant between CA and controls (*P* = .004). (Table 3).

#### Liver fibrosis, MRE-LSM, and noninvasive fibrosis scores of the Participants

The median MRE-LSM were 2.31 kPa (2.07-2.5) in AA, 2.29 kPa (2.11-2.41) in CA, and 2.17 kPa (1.97-2.43) in the control group. Three (21.4%) patients in AA, 4 (23.5%) patients in CA, and 2 (10.5%) in the control group had increased LSM. The groups were similar in terms of median MRE-LSM and frequency of increased LSM. Of the noninvasive fibrosis scores, the NFS, APRI, and FIB-4 were similar between groups, whereas AA had a higher median BARD score than the control group (*P* = .018) (Table 3).

### Correlation of Noninvasive Scores and MRI-PDFF and MRE-LSM

#### Active acromegaly, controlled acromegaly, and the control groups

There was a positive correlation between liver MRI-PDFF and all of the noninvasive scores in the control group, whereas only TyG showed significant positive correlation with the liver MRI-PDFF in the AA and CA groups (Table 4). None of the noninvasive fibrosis scores showed a significant correlation with MRE-LSM in both the control and CA group. However, APRI (*r* = 0.669, *P* = .009), FIB-4 (*r* = 0.666, *P* = .009), and NFS (*r* = 0.574, *P* = .032) showed a significant positive correlation with MRE-LSM in the AA group.

**Table 4.** Correlation between liver MRI-PDFF, noninvasive scores, and traditional NAFLD risk factors

Variable	Active acromegaly(n = 15)		Controlled acromegaly (n = 17)		Control group(n = 19)		Whole acromegaly group (n = 32)	
	Liver MRI-PDFF (%)		Liver MRI-PDFF (%)		Liver MRI-PDFF (%)		Liver MRI-PDFF (%)	
	Correlation coefficient (*r*)	*P* value	Correlation coefficient (*r*)	*P* value	Correlation coefficient (*r*)	*P* value	Correlation coefficient (*r*)	*P* value
VAI	0.254	.382	0.380	.132	**0**.**550**	.**015**	**0**.**508**	.**004**
HSI	0.197	.5	0.072	.784	**0**.**598**	.**007**	0.234	.204
FLI	−0.04	.893	0.446	.073	**0**.**730**	**<**.**001**	**0**.**482**	.**006**
TyG	**0**.**534**	.**049**	**0**.**593**	.**012**	**0**.**557**	.**013**	**0**.**675**	**<**.**001**
BMI (kg/m^2^)	−0.93	.753	0.077	.768	0.412	.079	0.163	.380
Waist circumference (cm)	−0.280	.332	0.122	.642	**0**.**525**	.**021**	0.203	.273
Waist/hip ratio	−0.225	.439	0.002	.994	**0**.**564**	.**012**	0.108	.564
C-reactive protein (mg/dL)	0.192	.620	−0.023	.929	**0**.**495**	.**031**	0.071	.731
HOMA-IR	0.313	.275	0.216	.405	**0**.**528**	.**02**	0.316	.089
Triglyceride (mg/dL)	0.496	.071	0.476	.053	**0**.**494**	.**032**	**0**.**625**	**<**.**001**
Visceral adipose tissue (cm^2^)	−0.432	.123	0.264	.306	**0**.**823**	**<**.**001**	**0**.**375**	.**037**

The boldface *P* values indicate statistical significance at the *P* ≤ .05 level.

Abbreviations: BMI, body mass index; FLI, fatty liver index; HOMA-IR, homeostasis model assessment index-insulin resistance; HSI, hepatic steatosis index; NAFLD, nonalcoholic fatty liver disease; TyG Index, triglyceride-glucose index; VAI, visceral adiposity index.

The correlations between BMI, waist circumference, waist/hip ratio, HOMA-IR, C-reactive protein, triglyceride, VAT, which are known to be traditional risk factors for NAFLD, and MRI-PDFF were examined. All these parameters (except BMI) showed a positive and significant correlation with liver MRI-PDFF in the control group. However, none of these parameters showed a meaningful correlation with the liver MRI-PDFF in both the AA and CA groups. At the same time, there was a positive correlation between BMI and liver MRI-PDFF in the control group and between triglycerides and liver MRI-PDFF in the AA and CA groups. Although these correlations were insignificant at the *P* < .05 level, they showed a tendency to be significant (Table 4).

#### Whole acromegaly group

After the separate evaluation of each group, we combined the AA and CA groups as a single “acromegaly group” to understand the general approach for the evaluation of the patients with acromegaly for NAFLD. In the acromegaly group, liver MRI-PDFF was positively correlated with TyG (*r* = 0.675, *P* < .001), VAI (*r* = 0.508, *P* = .004), and FLI (*r* = 0.482, *P* = .006), whereas there was no correlation with HSI. Receiver operating characteristic analysis of the noninvasive hepatosteatosis scores in patients with acromegaly are shown in Table 5. Of the noninvasive fibrosis scores, only APRI showed a modest positive correlation with MRE-LSM (*r* = 0.364, *P* = .044) in the acromegaly group.

**Table 5.** ROC analysis of noninvasive hepatosteatosis in acromegaly patients

Score	AUROC(% 95 GA)	Optimal Cutoff	Sensitivity (%)	Specificity (%)	PPV (%)	NPV (%)	*P* value
VAI	0.78 (0.61-0.96)	1.58	90	52.4	47.4	91.7	0.013
FLI	0.77 (0.60-0.94)	58	90	66.7	56.3	93.3	0.015
TyG	0.86 (0.73-0.99)	8.96	80	76	61.5	88.9	0.002

Abbreviations: AUROC, area under receiver operating curve; FLI, fatty liver index; NPV, negative predictive value; PPV, positive predictive value; TyG Index, triglyceride-glucose index; VAI, visceral adiposity index.

The correlation analysis showed that only the triglyceride level and VAT showed significant correlation with liver MRI-PDFF (Table 4).

**Figure 1.**
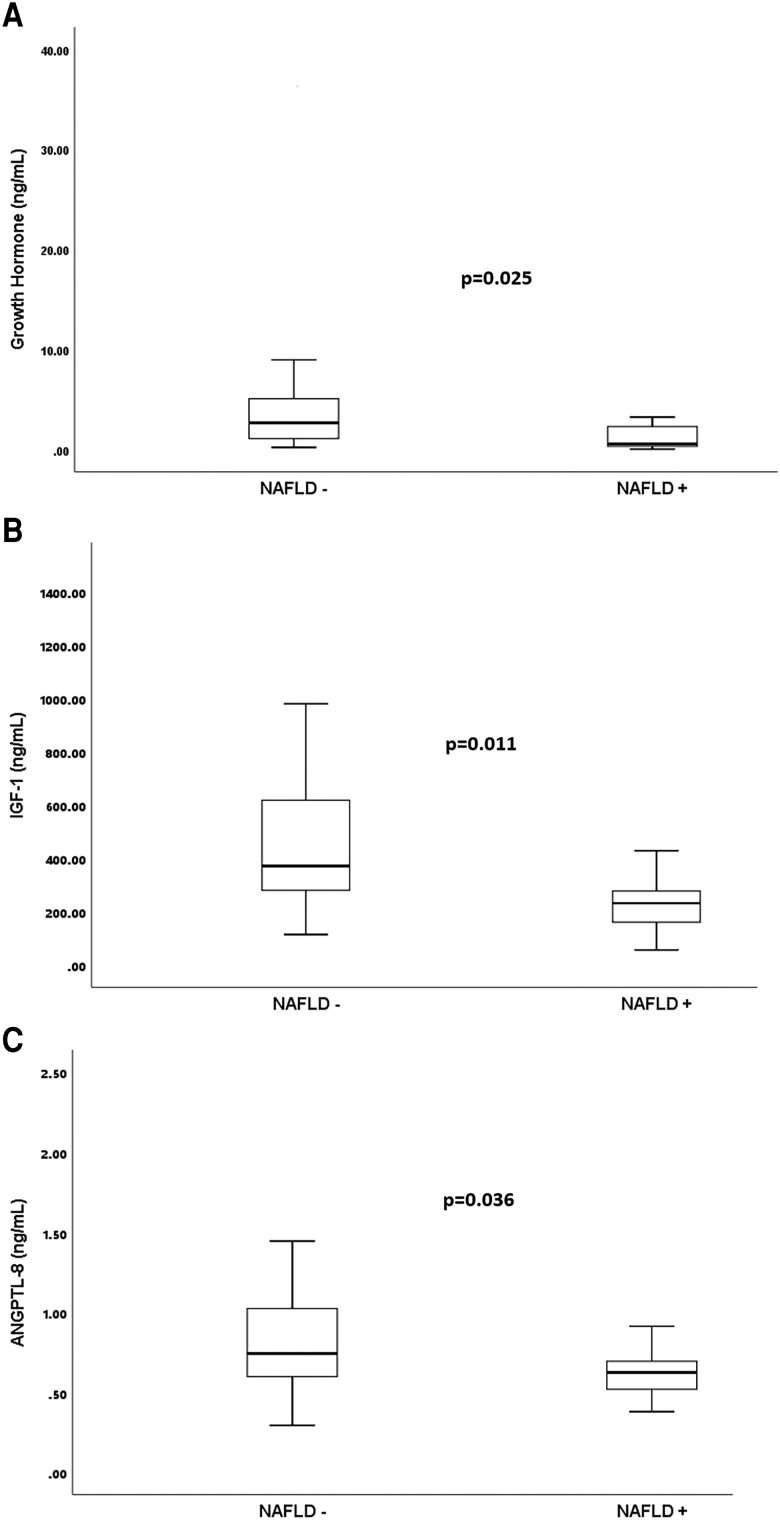
(A) Distribution of GH in those with and without NAFLD (B) Distribution of IGF-1 in those with and without NAFLD (C) Distribution of serum angiopoietin-like protein-8 in those with and without NAFLD ANGPTL-8, angiopoietin-like protein-8; NAFLD, nonalcoholic fatty liver disease; NAFLD-, absence of NAFLD; NAFLD+, presence of NAFLD.

Serum ANGPTL-8 levels, IGF-1, and GH were significantly lower in those with NAFLD in the acromegaly group (Fig. 1A–C).

#### Comparison of the new and previously defined cutoff values for noninvasive hepatosteatosis scores in the active and controlled patients with acromegaly

The risk of NAFLD in AA and CA patients according to the previously defined cutoff values in the nonacromegalic population and cutoff values we found in this study is shown in Supplementary Tables S1 and 2 (25). According to these results, all patients (n = 5) with severe adipose tissue dysfunction had NAFLD in the CA group. The 1.58 cutoff value we found for VAI was accurate in 6 (42.9%) of 14 patients in the AA group in the prediction of NAFLD diagnosis, whereas it was accurate in 12 (70.6%) of 17 patients in the CA group. In the AA group, FLI was correct in 10 (71.4%) of 14 patients, according to both the previously defined cutoff in the nonacromegalic population (if the indeterminate risk is also considered as low risk for NAFLD) and the cutoff we found. On the other hand, in the CA group, both cutoffs were working in 13 (76.5%) of 17 patients. For TyG, although the old cutoff was correct only in 6 (42.9%) of 14 patients in the AA group, it was working in 11 (78.6%) patients according to the new cutoff. In the CA group, the old value was correct in 12 (70.6%) patients, whereas the new value was correct in 13 (76.5%) patients for the prediction of NAFLD diagnosis.

## Discussion

This study found that AA patients have lower liver fat compared with CA patients. There was no correlation between liver fat ratio and traditional risk factors for NAFLD in the AA and CA groups. Noninvasive hepatosteatosis scores used in the nonacromegalic population were less sensitive and specific for patients with acromegaly. Moreover, their utility also differs between the AA and CA groups. We defined new cutoff values for the noninvasive hepatosteatosis scores; of these, FLI and TyG showed the best performance to diagnose NAFLD, whereas only TyG showed a significant correlation with liver fat ratio in both the AA and CA groups. The median ANGPTL-8, GH, and IGF-1 levels of the patients with NAFLD were lower than those without NAFLD in the whole acromegaly group.

The link between NAFLD and acromegaly is controversial because of the complicated metabolic effects of GH. Growth hormone increases gluconeogenesis and glycogenolysis in the muscle and liver. Its lipolytic activity also releases free fatty acids into the circulation. Insulin resistance and high free fatty acid levels are well-known risk factors for NAFLD (33, 34). Conversely, enhanced lipolysis may protect patients with acromegaly from developing NAFLD (3).

Despite severe insulin resistance, active patients with acromegaly were shown to have reduced hepatocellular lipid content measured by magnetic resonance spectroscopy. Moreover, individuals with acromegaly also exhibited an increased intrahepatic lipid ratio with biochemical control (4, 5). Our study used MRI-PDFF rather than magnetic resonance spectroscopy but found similar results. Although the primary mechanism underlying the lower liver fat content in active acromegaly is unknown, Fellinger et al suggested that it may be related to increased hepatic ATP production (3).

Although NAFLD is common in the community, the true prevalence of NAFLD in patients with acromegaly is unknown. In a German study in which 80% of the subjects had well-controlled acromegaly, ultrasonography (US) revealed NAFLD in 66% of the participants (35). Ciresi et al found that 61% of patients with active acromegaly at diagnosis had NAFLD determined by the US. Although 58% of individuals controlled their acromegaly after 12 months of therapy, the same participants still had NAFLD (9). In our research, 32% of patients with acromegaly had NAFLD, which was lower than in European studies. This result may be due to demographic differences or because we used MRI, which is more sensitive in detecting NAFLD. We also divided patients with acromegaly into 2 groups based on their disease control. NAFLD was not found in any of the patients with AA but in 58% of the patients with CA. These data suggest that NAFLD can progress differently in AA and CA patients, and it may be best to study these 2 groups separately.

We also evaluated the diagnostic capacities of the noninvasive hepatosteatosis and fibrosis scores in patients with acromegaly for the first time. Although all of the noninvasive scores showed a positive correlation with liver fat in the control group, only TyG was correlated with liver fat ratio in both the AA and CA patients. When we evaluated the whole acromegaly group together, we found that VAI, FLI, and TyG indices were diagnostically adequate, although TyG had the greatest area under the ROC. However, HSI was insufficient for identifying NAFLD in the acromegalic individuals. The optimal cutoff values for FLI, VAI, and TyG index for diagnosing NAFLD in patients with acromegaly were 58, 1.58, and 8.96, respectively. The higher negative predictive values of the scores indicate that these scores could result in false positives. Although these cutoff values are important because they are the first to be reported, they need to be verified by studies with a larger sample size of patients with acromegaly. Moreover, our study indicated that the NAFLD process and the diagnostic capacity of the noninvasive scores differ regarding disease control. They should be verified differently in AA and CA patients in larger cohorts. None of these scores were validated in patients with acromegaly before. However, Ciresi et al used the HSI score to evaluate hepatosteatosis severity in patients with acromegaly. Whereas 83% of the active patients with acromegaly had a high HSI score before therapy, this proportion dropped to 45% after treatment (9). However, our results showed no correlation between HSI and liver MRI-PDFF in patients with acromegaly. Therefore, the HSI score may not be appropriate for measuring hepatosteatosis severity in patients with acromegaly. Ciresi et al also found that HSI scores of patients with acromegaly reduced with disease management (9). Our study contradicts this conclusion because controlled patients with acromegaly had more liver fat.

Although FLI, HSI, and VAI scores use BMI as a scoring system variable, TyG does not. This could explain why TyG gives a better performance in people with acromegaly. BMI is one of the most widely used measures of adiposity and is one of the most significant risk factors for NAFLD (36, 37). Nonetheless, the rise in BMI in patients with acromegaly is mainly related to the expansion of the soft and bone tissue and visceromegaly. Given the lipolytic effect of the GH, BMI may not be a valuable metric for evaluating obesity in patients with acromegaly (33, 38). In our study, liver MRI-PDFF and BMI were not correlated in patients with acromegaly, which implies that BMI-related scores and risk factors used in the nonacromegalic population may not be appropriate in acromegaly. Moreover, the AA, CA, and control groups were similar in terms of BMI in our study. However, waist circumference was higher in the CA group and VAT was lower in the AA group. The difference in liver fat ratio between the AA and CA groups may be due to the difference in visceral adiposity. This finding also supports that BMI is not a direct indicator of central adiposity in patients with acromegaly. Besides BMI, waist circumference, waist/hip ratio, C-reactive protein, HOMA-IR, triglyceride, VAT, and liver MRI-PDFF were positively correlated in the control group, but not in the AA and CA groups. These results show that classical risk factors may not be adequate for evaluating NAFLD risk, and new risk factors should be determined to evaluate NAFLD risk in patients with acromegaly. On the other hand, triglyceride levels showed a significant correlation with liver fat in the whole acromegaly group. Moreover, the correlation between triglyceride and liver MRI-PDFF also tended to be significant in both the AA and CA groups. This result could support that effective triglyceride control in patients with acromegaly might be an important step for preventing NAFLD, yet it should be validated with longitudinal studies.

Pegvisomant therapy has also been demonstrated to enhance hepatic steatosis (39). Only 2 patients were getting pegvisomant in our research. The duration and cumulative dosages were similar. Hepatosteatosis was not identified in the patient with active disease, but moderate hepatosteatosis was reported in the patient with controlled disease. Because the patients’ baseline liver fat ratio is unknown and only 1 patient in each group received pegvisomant medication, no conclusion can be made about the relationship between pegvisomant treatment and NAFLD in this study. That the patient in the AA group did not have NAFLD, whereas the patient in the CA group did, could show that disease management may have an important impact on the presence of NAFLD.

We found that GH and IGF-1 levels were lower in those with NAFLD than those without NAFLD in the patients with acromegaly. Individuals with GH deficiency are known to be more prone to develop hepatosteatosis (40, 41). Furthermore, it has been demonstrated that patients with NAFLD have a lower baseline and stimulated GH levels (42, 43). Two small studies have shown that recombinant GH therapy may improve fatty liver (44, 45). In our study, NAFLD was not found in any of the AA patients, despite higher insulin resistance. All these data support the idea that GH may protect against fatty liver disease.

Liver fibrosis is a major complication of NAFLD. Although biopsy is the gold standard, MRE and US elastography can also evaluate liver stiffness. Several studies have demonstrated that NFS, FIB-4, APRI, and BARD may be used to determine NAFLD patients with a high risk of fibrosis (17, 18). In our study, no fibrosis score were significant in predicting fibrosis in the control and the CA group. This is most likely because of our sample's lack of individuals with severe fibrosis. Although GH's increased influence on collagen production may lead to fibrosis (46), liver fibrosis in individuals with acromegaly has not been well examined. In our research, a sensitive technique, MRE, was used, and no significant differences were seen in the median MRE-LSM and prevalence of increased LSM between the groups. Interestingly, NFS, APRI, and FIB-4 showed a strong correlation with liver stiffness measurement in the AA patients. In larger cohorts, liver fibrosis and the validity of noninvasive fibrosis scores should be investigated in patients with acromegaly.

ANGPTL-8 is a crucial protein contributing to lipid and glucose metabolism through its action on lipoprotein lipase. It is associated with an altered metabolic profile (47-49). Human and animal research and meta-analyses indicated that ANGPTL-8 is elevated in patients with NAFLD and can be used as a marker for NAFLD patients. Elevated levels of ANGPTL-8 were also correlated with the severity of NAFLD (20, 21, 50). Alternatively, some also hypothesized that the rise in serum ANGPTL-8 level in metabolic illnesses might be a compensatory response to the increased insulin resistance (51). Interestingly, non-NAFLD acromegalic patients showed higher serum ANGPTL-8 levels than patients with acromegaly with NAFLD in our study. Despite this being the first study evaluating the relation between the NAFLD and ANGPTL-8 in patients with acromegaly, this result supports the hypothesis that ANGPTL-8 may elevate as a compensatory response to the disturbed metabolic profile rather than being a cause in the pathophysiology of NAFLD.

This study has some limitations and strengths. Because of the rarity of acromegaly, the relatively small sample size may have caused a type II error. In addition, heterogeneity of the patients with acromegaly regarding their drugs can affect the results. Because of the cross-sectional design, we did not know the patients’ baseline values; therefore, we could not establish a causal relation. Moreover, the noninvasive scores we used to quantify liver parameters have not been validated in patients with acromegaly with liver biopsy or liver fat in prior studies. Therefore, we used these scores based on studies done with nonacromegalic populations. We excluded the individuals with a previous suspicion of NAFLD in the control group. These could limit the representative value of our control group and may affect the comparison analyses, especially between the control group and the CA group. On the other hand, because dysglycemia is very common in acromegaly, we included patients with prediabetes and diabetes mellitus in the acromegaly group but not in the control group. This could add a significant bias, given that diabetes mellitus is closely associated with NAFLD. However, lower intrahepatic fat despite higher diabetes mellitus prevalence in patients with active acromegaly also strengthened our results. Additionally, we could not eliminate the potential effects of the antidiabetic medications of the acromegaly patients on the results. The study also possesses some strengths. First, the acromegaly group was matched with its control group based on age, gender, and BMI. The liver MRI-PDFF, MRE-LSM, and VAT were all assessed using MRI, a very sensitive and specific approach. The strongest part of this study is that many previously unexplored topics including evaluation of noninvasive scores and the relationship between ANGPTL-8 and hepatic steatosis in patients with acromegaly are presented for the first time.

## Conclusion

In conclusion, high GH levels seem to be protective against fatty liver in patients with active acromegaly, even if the metabolic profile is impaired. No relation was observed between liver MRI-PDFF and conventional risk factors in patients with acromegaly; larger population studies are needed to establish the optimal risk factors for the existence of NAFLD in patients with acromegaly. The strongest connection was observed between liver MRI-PDFF and TyG index among the noninvasive hepatosteatosis scores in patients with acromegaly. In individuals with acromegaly, it seems inappropriate to use noninvasive scores in which BMI is variable. However, one of the most important results of our study is that the NAFLD process can progress differently and the performance of the noninvasive scores can differ in patients with active and controlled acromegaly. Therefore, it may be more appropriate to study NAFLD risk factors separately in active and controlled patients with acromegaly in larger populations. ANGPTL-8 levels were lower in patients with acromegaly with NAFLD, suggesting that high levels represent a reaction to insulin resistance rather than being a cause of NAFLD. In patients with acromegaly, treatment planning at a level that will not cause GH deficiency while maintaining disease control may be beneficial in terms of reducing NAFLD development.

## Data Availability

Some or all datasets generated during and/or analyzed during the current study are not publicly available but are available from the corresponding author on reasonable request.

## Abbreviations

AA: active acromegaly
ANGPTL-8: angiopoietin-like protein-8
APRI: aspartate aminotransferase/platelet ratio index
BMI: body mass index
CA: controlled acromegaly
FIB-4: fibrosis-4 score
FLI: fatty liver index
HOMA-IR: homeostasis model assessment for insulin resistance
HSI: hepatic steatosis index
LSM: liver stiffness measurement
MRE: magnetic resonance elastography
MRI: magnetic resonance imaging
NAFLD: nonalcoholic fatty liver disease
NFS: nonalcoholic fatty liver disease fibrosis score
PDFF: proton density fat fraction
ROI: region of interest
TyG: triglyceride-glucose index
US: ultrasonography
VAI: visceral adiposity index
VAT: visceral adipose tissue

## References

[1] Ruissen MM , Mak AL, Beuers U, Tushuizen ME, Holleboom AG. Non-alcoholic fatty liver disease: a multidisciplinary approach towards a cardiometabolic liver disease. Eur J Endocrinol. 2020;183(3):R57‐R73. 32508312 10.1530/EJE-20-0065

[2] Marino L , Jornayvaz FR. Endocrine causes of nonalcoholic fatty liver disease. World J Gastroenterol. 2015;21(39):11053‐11076. 26494962 10.3748/wjg.v21.i39.11053 PMC4607905

[3] Fellinger P , Wolf P, Pfleger L, et al Increased ATP synthesis might counteract hepatic lipid accumulation in acromegaly. JCI Insight. 2020;5(5):e134638. 10.1172/jci.insight.134638 PMC7141383 32106111

[4] Winhofer Y , Wolf P, Krššák M, et al No evidence of ectopic lipid accumulation in the pathophysiology of the acromegalic cardiomyopathy. J Clin Endocrinol Metab. 2014;99(11):4299‐4306. 25148232 10.1210/jc.2014-2242

[5] Bredella MA , Schorr M, Dichtel LE, et al Body composition and ectopic lipid changes with biochemical control of acromegaly. J Clin Endocrinol Metab. 2017;102(11):4218‐4225. 28945897 10.1210/jc.2017-01210 PMC6283448

[6] Arlien-Søborg MC , Madsen MA, Dal J, et al Ectopic lipid deposition and insulin resistance in patients with GH disorders before and after treatment. Eur J Endocrinol. 2023;188(1):78‐85. 10.1093/ejendo/lvac014 36651164

[7] Reyes-Vidal CM , Mojahed H, Shen W, et al Adipose tissue redistribution and ectopic lipid deposition in active acromegaly and effects of surgical treatment. J Clin Endocrinol Metab. 2015;100(8):2946‐2955. 26037515 10.1210/jc.2015-1917 PMC4524994

[8] Kuker AP , Shen W, Jin Z, Chen J, Bruce JN, Freda PU. Long-term outcome of body composition, ectopic lipid, and insulin resistance changes with surgical treatment of acromegaly. J Endocr Soc. 2023;7(5):bvad028. 10.1210/jendso/bvad028 PMC10008673 36922916

[9] Ciresi A , Guarnotta V, Campo D, Giordano C. Hepatic Steatosis Index in acromegaly: correlation with insulin resistance regardless of the disease control. Int J Endocrinol. 2018;2018:5421961. 10.1155/2018/5421961 PMC6313980 30662461

[10] Freda PU . The acromegaly lipodystrophy. Front Endocrinol (Lausanne). 2022;13:933039. 10.3389/fendo.2022.933039 PMC9513226 36176462

[11] Dulai PS , Sirlin CB, Loomba R. MRI and MRE for non-invasive quantitative assessment of hepatic steatosis and fibrosis in NAFLD and NASH: clinical trials to clinical practice. J Hepatol. 2016;65(5):1006‐1016. 27312947 10.1016/j.jhep.2016.06.005 PMC5124376

[12] Fedchuk L , Nascimbeni F, Pais R, Charlotte F, Housset C, Ratziu V. Performance and limitations of steatosis biomarkers in patients with nonalcoholic fatty liver disease. Aliment Pharmacol Ther. 2014;40(10):1209‐1222. 25267215 10.1111/apt.12963

[13] Bedogni G , Bellentani S, Miglioli L, et al The fatty liver index: a simple and accurate predictor of hepatic steatosis in the general population. BMC Gastroenterol. 2006;6(1):33. 17081293 10.1186/1471-230X-6-33 PMC1636651

[14] Lee JH , Kim D, Kim HJ, et al Hepatic steatosis index: a simple screening tool reflecting nonalcoholic fatty liver disease. Dig Liver Dis. 2010;42(7):503‐508. 19766548 10.1016/j.dld.2009.08.002

[15] Xu C , Ma Z, Wang Y, et al Visceral adiposity index as a predictor of NAFLD: a prospective study with 4-year follow-up. Liver Int. 2018;38(12):2294‐2300. 30099825 10.1111/liv.13941

[16] Zhang S , Du T, Zhang J, et al The triglyceride and glucose index (TyG) is an effective biomarker to identify nonalcoholic fatty liver disease. Lipids Health Dis. 2017;16(1):15. 28103934 10.1186/s12944-017-0409-6 PMC5248473

[17] Subasi CF , Aykut UE, Yilmaz Y. Comparison of noninvasive scores for the detection of advanced fibrosis in patients with nonalcoholic fatty liver disease. Eur J Gastroenterol Hepatol. 2015;27(2):137‐141. 25486027 10.1097/MEG.0000000000000255

[18] Chen X , Goh GB, Huang J, et al Validation of non-invasive fibrosis scores for predicting advanced fibrosis in metabolic-associated fatty liver disease. J Clin Transl Hepatol. 2022;10(4):589‐594. 36062270 10.14218/JCTH.2021.00311 PMC9396333

[19] Hong BS , Liu J, Zheng J, et al Angiopoietin-like protein 8/betatrophin correlates with hepatocellular lipid content independent of insulin resistance in non-alcoholic fatty liver disease patients. J Diabetes Investig. 2018;9(4):952‐958. 10.1111/jdi.12792 PMC6031491 29266821

[20] Ke Y , Liu S, Zhang Z, Hu J. Circulating angiopoietin-like proteins in metabolic-associated fatty liver disease: a systematic review and meta-analysis. Lipids Health Dis. 2021;20(1):55. 34034750 10.1186/s12944-021-01481-1 PMC8152125

[21] Lee Y-H , Lee S-G, Lee CJ, et al Association between betatrophin/ANGPTL8 and non-alcoholic fatty liver disease: animal and human studies. Sci Rep. 2016;6(1):24013. 27045862 10.1038/srep24013 PMC4820743

[22] Khaire SS , Gada JV, Varthakavi PK, Bhagwat NM. Prevalence and predictors of abnormal glucose tolerance and its resolution in acromegaly: single centre retrospective study of 90 cases. Growth Horm IGF Res. 2021;59:101394. 33984540 10.1016/j.ghir.2021.101394

[23] Khan SA , Ram N, Masood MQ. Patterns of abnormal glucose metabolism in acromegaly and impact of treatment modalities on glucose metabolism. Cureus. 2021;13(3):e13852. 33859902 10.7759/cureus.13852 PMC8038903

[24] Matthews DR , Hosker J, Rudenski A, Naylor B, Treacher D, Turner R. Homeostasis model assessment: insulin resistance and β-cell function from fasting plasma glucose and insulin concentrations in man. Diabetologia. 1985;28(7):412‐419. 3899825 10.1007/BF00280883

[25] Eroğlu İ , Iremli BG, Idilman IS, et al Nonalcoholic fatty liver disease, Liver fibrosis, and utility of noninvasive scores in patients with acromegaly. J Clin Endocrinol Metab. 2024;109(1):e119-e129. 10.1210/clinem/dgad490 PMC10735300 37590020

[26] Amato MC , Giordano C. Visceral adiposity index: an indicator of adipose tissue dysfunction. Int J Endocrinol. 2014;2014:730827. 10.1155/2014/730827 PMC4009335 24829577

[27] Angulo P , Hui JM, Marchesini G, et al The NAFLD fibrosis score: a noninvasive system that identifies liver fibrosis in patients with NAFLD. Hepatology. 2007;45(4):846‐854. 17393509 10.1002/hep.21496

[28] Lin ZH , Xin YN, Dong QJ, et al Performance of the aspartate aminotransferase-to-platelet ratio index for the staging of hepatitis C-related fibrosis: an updated meta-analysis. Hepatology. 2011;53(3):726‐736. 21319189 10.1002/hep.24105

[29] Harrison SA , Oliver D, Arnold HL, Gogia S, Neuschwander-Tetri BA. Development and validation of a simple NAFLD clinical scoring system for identifying patients without advanced disease. Gut. 2008;57(10):1441‐1447. 18390575 10.1136/gut.2007.146019

[30] Sterling RK , Lissen E, Clumeck N, et al Development of a simple noninvasive index to predict significant fibrosis in patients with HIV/HCV coinfection. Hepatology. 2006;43(6):1317‐1325. 16729309 10.1002/hep.21178

[31] Kühn JP , Meffert P, Heske C, et al Prevalence of fatty liver disease and hepatic iron overload in a northeastern German population by using quantitative MR imaging. Radiology. 2017;284(3):706‐716. 28481195 10.1148/radiol.2017161228 PMC5565690

[32] Swinscow TDV , Campbell MJ. Statistics at square one: Bmj London. 2002.

[33] Møller N , Jørgensen JO. Effects of growth hormone on glucose, lipid, and protein metabolism in human subjects. Endocr Rev. 2009;30(2):152‐177. 19240267 10.1210/er.2008-0027

[34] Rizza RA , Mandarino LJ, Gerich JE. Effects of growth hormone on insulin action in man. Mechanisms of insulin resistance, impaired suppression of glucose production, and impaired stimulation of glucose utilization. Diabetes. 1982;31(8 Pt 1):663‐669. 6761205 10.2337/diab.31.8.663

[35] Koutsou-Tassopoulou A , Papapostoli-Sklavounou I, Krawczyk M, et al Hepatic steatosis in patients with acromegaly. Endocrinol Diabetes Metab. 2019;2(4):e00090. 31592448 10.1002/edm2.90 PMC6775446

[36] Shah NR , Braverman ER. Measuring adiposity in patients: the utility of body mass index (BMI), percent body fat, and leptin. PLoS One. 2012;7(4):e33308. 22485140 10.1371/journal.pone.0033308 PMC3317663

[37] Fan R , Wang J, Du J. Association between body mass index and fatty liver risk: a dose-response analysis. Sci Rep. 2018;8(1):15273. 30323178 10.1038/s41598-018-33419-6 PMC6189125

[38] Lugo G , Pena L, Cordido F. Clinical manifestations and diagnosis of acromegaly. Int J Endocrinol. 2012;2012:540398. 22518126 10.1155/2012/540398 PMC3296170

[39] Madsen M , Krusenstjerna-Hafstrøm T, Møller L, et al Fat content in liver and skeletal muscle changes in a reciprocal manner in patients with acromegaly during combination therapy with a somatostatin analog and a GH receptor antagonist: a randomized clinical trial. J Clin Endocrinol Metab. 2012;97(4):1227‐1235. 22298804 10.1210/jc.2011-2681

[40] Nishizawa H , Iguchi G, Murawaki A, et al Nonalcoholic fatty liver disease in adult hypopituitary patients with GH deficiency and the impact of GH replacement therapy. Eur J Endocrinol. 2012;167(1):67‐74. 22535644 10.1530/EJE-12-0252

[41] Ichikawa T , Hamasaki K, Ishikawa H, Ejima E, Eguchi K, Nakao K. Non-alcoholic steatohepatitis and hepatic steatosis in patients with adult onset growth hormone deficiency. Gut. 2003;52(6):914. 10.1136/gut.52.6.914 PMC1773673 12740357

[42] Dichtel LE , Corey KE, Haines MS, et al The GH/IGF-1 axis is associated with intrahepatic lipid content and hepatocellular damage in overweight/obesity. J Clin Endocrinol Metab. 2022;107(9):e3624‐e3e32. 35779256 10.1210/clinem/dgac405 PMC9387707

[43] Xu L , Xu C, Yu C, et al Association between serum growth hormone levels and nonalcoholic fatty liver disease: a cross-sectional study. PLoS One. 2012;7(8):e44136. 22952901 10.1371/journal.pone.0044136 PMC3432067

[44] Bartsch L , Bredella M, Chicote ML, et al OR27-2 growth hormone reduces hepatic steatosis, inflammation and fibrosis in adults with overweight/obesity and nonalcoholic fatty liver disease. J Endocr Soc. 2022;6(Supplement_1):A525-A.

[45] Xue J , Liang S, Ma J, Xiao Y. Effect of growth hormone therapy on liver enzyme and other cardiometabolic risk factors in boys with obesity and nonalcoholic fatty liver disease. BMC Endocr Disord. 2022;22(1):49. 35216556 10.1186/s12902-022-00967-y PMC8881210

[46] Kopchick JJ , Basu R, Berryman DE, Jorgensen JOL, Johannsson G, Puri V. Covert actions of growth hormone: fibrosis, cardiovascular diseases and cancer. Nat Rev Endocrinol. 2022;18(9):558‐573. 35750929 10.1038/s41574-022-00702-6 PMC9703363

[47] Yang S , Jiao X, Huo X, et al Association between circulating full-length angiopoietin-like protein 8 and non-high-density lipoprotein cholesterol levels in Chinese non-diabetic individuals: a cross-sectional study. Lipids Health Dis. 2018;17(1):161. 30021605 10.1186/s12944-018-0802-9 PMC6052512

[48] Fu Z , Berhane F, Fite A, Seyoum B, Abou-Samra AB, Zhang R. Elevated circulating lipasin/betatrophin in human type 2 diabetes and obesity. Sci Rep. 2014;4(1):5013. 24852694 10.1038/srep05013 PMC5381405

[49] Guo C , Zhao Z, Deng X, Chen Z, Tu Z, Yuan G. Regulation of angiopoietin-like protein 8 expression under different nutritional and metabolic status. Endocr J. 2019;66(12):1039‐1046. 31631098 10.1507/endocrj.EJ19-0263

[50] Mele C , Crinò A, Fintini D, et al Angiopoietin-like 8 (ANGPTL8) as a potential predictor of NAFLD in paediatric patients with Prader-Willi syndrome. J Endocrinol Invest. 2021;44(7):1447‐1456. 33067796 10.1007/s40618-020-01444-w PMC8195791

[51] Guo C , Wang C, Deng X, He J, Yang L, Yuan G. Angptl8 in metabolic homeostasis: more friend than foe? Open Biol. 2021;11(9):210106. 10.1098/rsob.210106 PMC8478524 34582711

